# Pravastatin‐induced changes in expression of long non‐coding and coding RNAs in endothelial cells

**DOI:** 10.14814/phy2.14661

**Published:** 2020-12-28

**Authors:** Shweta Singh, Hien C. Nguyen, Mehroz Ehsan, David C. R. Michels, Priyanka Singh, Mohammad Qadura, Krishna K. Singh

**Affiliations:** ^1^ Department of Chemical and Biochemical Engineering Schulich School of Medicine and Dentistry University of Western Ontario London ON Canada; ^2^ Department of Medical Biophysics Schulich School of Medicine and Dentistry University of Western Ontario London ON Canada; ^3^ Department of Anatomy and Cell Biology Schulich School of Medicine and Dentistry University of Western Ontario London ON Canada; ^4^ Schulich School of Medicine and Dentistry University of Western Ontario London ON Canada; ^5^ Vascular Surgery Keenan Research Centre for Biomedical Science and Li Ka Shing Knowledge Institute of St. Michael’s Hospital Toronto ON Canada; ^6^ Institute of Medical Science University of Toronto Toronto ON Canada; ^7^ Pharmacology and Toxicology University of Toronto Toronto ON Canada

**Keywords:** coronary artery disease, endothelial dysfunction, lncRNA, pravastatin

## Abstract

**Objective:**

Atherosclerosis is the main cause of the cardiovascular disease (CVD). Elevated blood cholesterol and inflammation of the endothelium are two major mechanisms contributing to the establishment of atherosclerotic plaques. Statins, such as pravastatin, are blood‐cholesterol lowering drugs commonly prescribed for patients with or at risk for CVDs. In addition to lowering blood cholesterols, statins have recently been shown to improve endothelial function in both hyper‐ and normocholesterolemic patients with atherosclerosis. To understand the molecular mechanisms underlying the endothelial function improvement by statins, we assessed the RNA profile of pravastatin‐treated endothelial cells, particularly their mRNAs and long non‐coding RNAs (lncRNAs).

**Methods:**

Human umbilical vein endothelial cells (HUVECs) treated with pravastatin (10 µM) for 24 hr were profiled for lncRNAs and mRNAs using the Arraystar Human lncRNA Expression Microarray V3.0.

**Results:**

Of the 30,584 different lncRNAs screened, 95 were significantly upregulated, while 86 were downregulated in HUVECs responding to pravastatin. LINC00281 and BC045663 were the most upregulated (~8‐fold) and downregulated (~3.5‐fold) lncRNAs, respectively. Of the 26,106 different mRNAs screened in the pravastatin‐treated HUVEC samples, 190 were significantly upregulated, while 90 were downregulated. Assigning the differentially expressed genes by bioinformatics into functional groups revealed their molecular signaling involvement in the following physiological processes: osteoclast differentiation, Rap1 signaling pathway, hematopoiesis, immunity, and neurotrophin signaling pathway.

**Conclusions:**

This is the first lncRNA and mRNA expression profiling of pravastatin‐mediated changes in human endothelial cells. Our results reveal potential novel targets and mechanisms for pravastatin‐mediated vascular protection in atherosclerosis.

## Graphical abstract

First lncRNA and mRNA expression profiling of pravastatin‐mediated changes in human endothelial cells. We provide the basis for mechanisms associated with pravastatin to improve endothelial function.

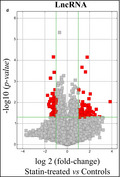

## 1 INTRODUCTION

Evidence from epidemiological, genetics and basic science studies associate elevated levels of plasma cholesterol with increased risk of atherosclerosis (Robinson et al., (2018); Kannel et al., 1964). Elevated cholesterol levels, also known as hypercholesterolemia, lead to endothelial dysfunction, which is now known to play a critical role in the majority of cardiovascular diseases including coronary artery disease (CAD) (Libby, 2001). The anatomical location of the endothelium enables it to sense hemodynamic and chemical changes within the blood to maintain vascular homeostasis by regulating the balance between vasoconstriction and vasodilatation. LDL (low‐density lipoprotein) cholesterol disturbs this homeostasis *via* several means that include the impairing production of the vasodilator nitric oxide (NO) by inactivating endothelial nitric oxide synthase (eNOS) in endothelial cells (ECs), disrupting the vasomotor tone (Hermida & Balligand, 2014; Noor et al., 2007; Steinberg et al., 1997). Accordingly, cholesterol‐lowering drugs have been shown to reduce the burden of CAD, in part due to improved endothelial vasomotor tone and reduced coronary artery dilatation (Anderson et al., 1995; Ward et al., 2019).

Statins are a class of drugs designed to lower blood cholesterol levels. While different statins vary in bioavailability and half‐life, they are widely prescribed for hypercholesterolemic patients who are at risk for cardiovascular diseases (CVDs) (Meor Anuar Shuhaili et al., 2017). The statin agents antagonize and inhibit the enzyme hepatic 3‐Hydroxy‐3‐Methylglutaryl‐CoA Reductase (HMG‐CoA reductase), which is normally responsible for an early rate‐limiting step in the biosynthesis of cholesterol and LDL. There are six statin drugs in use, including pitavastatin, atorvastatin, rosuvastatin, simvastatin, fluvastatin, and pravastatin. Except pravastatin, all other statins are metabolized by the cytochrome p450 group of enzymes and any drug interference with these enzymes can lead to an enhanced level of statin, thereby increasing the risk for toxicity (Ramkumar et al., 2016). Pravastatin has the least drug interaction among other statins and is the preferred statin for patients on immunosuppressants or protease inhibitors (Ramkumar et al., 2016). By blocking cholesterol synthesis in the liver, statins, such as pravastatin, not only reduce plasma LDL levels but also upregulate the hepatic LDL receptors, encouraging the liver to uptake and clear blood cholesterols (Pinal‐Fernandez et al., 2018). A lower level of plasma LDL is associated with a reduced risk for CVDs. Recently, a new perspective has emerged suggesting that statins‐mediated CVD risk reduction is not solely due to the lowering of blood cholesterols. Statins have been shown to restore or improve many aspects of endothelial function, which includes statins upregulating eNOS expression and activity through preventing the post‐translational modification of Rho and activating protein kinase Akt (Kureishi et al., 2000; Rikitake & Liao, 2005). In addition, potent vasoconstrictors dysregulated in atherosclerosis, such as endothelin‐1 and angiotensin‐II, were shown to be attenuated by statin treatments (Hernandez‐Perera et al., 1998; Ichiki et al., 2001). Statins can also control the vascular oxidative level and mitigate the induction of inflammatory mechanisms by downregulating endothelial inflammatory markers (Niwa et al., 1996; Rezaie‐Majd et al., 2002; Wassmann et al., 2001), thereby decreasing leukocyte recruitment.

Another new prospect of regulating endothelial function involves long‐non‐coding RNAs (lncRNAs). These transcripts are >200 nucleotides long and, although not as conserved, are transcribed like protein‐coding transcripts, similarly polyadenylated and capped, but are devoid of open reading frames (ORFs) (Djebali et al., 2012; Ulitsky & Bartel, 2013). Functionally, most lncRNAs interact with nuclear chromatin‐modifying ribonucleoprotein complexes as ligands and guide them through complementary base‐pairing to the targeted genomic sequences for epigenetic regulation. LncRNA’s mode of action is also characterized by whether the target sequence is within the vicinity of the lncRNA’s gene (*cis‐*acting) or not (*trans‐*acting) (Rinn et al., 2007). Previously regarded as transcriptional noise due to their low expression and poor evolutionary conservation (Hangauer et al., 2013). LncRNAs have recently emerged as potential molecular determinants of a wide range of diseases (Batista & Chang, 2013; Wapinski & Chang, 2011). Increasing evidence suggests the involvement of lncRNAs in a variety of cellular functions, including survival, proliferation, migration, invasion, angiogenesis and differentiation (Beltran et al., 2008; Fiedler et al., 2015; Guenther et al., 2007; Rinn et al., 2007; Wahlestedt, 2013; Zhao et al., 2008). Indeed, several lncRNAs have been notably characterized in cardiovascular pathophysiology: metastasis‐associated lung adenocarcinoma transcript 1 (MALAT1), one of the more conserved and abundant lncRNAs (Zhang et al., 2012) and Tie‐1‐AS were shown to regulate endothelial function (Wapinski & Chang, 2011; Zhao et al., 2008). Antisense noncoding RNA gene at the INK4 locus (ANRIL) and Apolipoprotein A1 antisense transcript (APOA1‐AS) were shown to have crucial roles in atherosclerosis (Holdt et al., 2013; Lund‐Katz & Phillips, 2010).

Given that statins and lncRNAs have been shown to impact endothelial function and cardiovascular status, it remains to be understood whether the endothelial processes regulated by lncRNAs are connected to statin's actions on ECs. Therefore, we aim to profile the variations in lncRNAs and mRNAs expression upon pravastatin treatment in ECs. Our approach would identify novel lncRNA and mRNA targets and associated pathways affected by pravastatin in ECs, thereby providing insights into the mechanisms behind pravastatin‐associated cardiovascular protection. This is the first transcriptome profiling of pravastatin‐mediated changes in human ECs.

## 2 MATERIALS AND METHODS

### 2.1 Cell culture and treatment

Human umbilical vein ECs (Pooled HUVECs, Lonza) were grown in the EC growth medium supplemented with growth factors, 5% fetal bovine serum (FBS), and antibiotics (EGM^TM^‐2 Bulletkit^TM^; Lonza) at 37^o^C and 5% CO_2_. Passage 4–6 HUVECs were used for the experiments. Cells were (60%–70%) confluent and were starved over‐night in MCDB‐131 basal medium with 1% FBS, followed by treatment with pravastatin sodium salt hydrate (10 µM; Sigma) (Abe et al., 2006; Panczel et al., 2019) in the same MCDB‐131 basal medium with 1% FBS for 24 hr. Controls were treated with vehicle phosphate‐buffered saline. To validate the expression level of top up‐ or downregulated lncRNAs and mRNAs, HUVECs were cultured and treated as previously described.

### 2.2 RNA extraction

HUVEC cultures were washed with ice‐cold PBS and then TRIzol^TM^ (Invitrogen) was directly added and incubated for 10 min at room temperature to extract total RNAs from HUVECs as instructed by the manufacturer. RNA was quantified using the NanoDrop ND‐1000 spectrophotometer and confirmed for integrity by standard denaturing agarose gel electrophoresis. Validation was performed for selected top up‐ or downregulated lncRNAs and mRNAs by extracting total RNA as previously described (Singh, Nguyen, et al., 2020). Later, cDNA was synthesized using 1μg of total RNA with the QuantiTect® Reverse Transcription kit (Qiagen) according to the manufacturer's instructions. Quantitative PCR (qPCR) was performed using primers for lncRNAs RP11‐469H8.6, LINC00281, BC045663, RP11‐791G16.2 and AC009948.5, and mRNAs DMKN, PIEGO2, APOLD1, ABI1, SND1, TMED2, SCAF8, SNX10, and GAPDH (Murugavel et al., 2018) (Table 1) using SYBR Select Master Mix (Applied Biosystems) and QuantStudio 3 Real‐Time PCR System (Applied Biosystems) (*N* = 3 in triplicates for both conditions). Fold‐change gene expression was calculated by the 2^‐ΔΔCt^ method, and the expression differences between the two groups were calculated using Student's *t* test. Validation data are presented as mean ± *SD*. A p‐value of less than 0.05 was considered significant.

**Table 1:** Primers used to perform qPCR

Nr	Gene symbol	Forward Primer ( 5′ to 3′)	Reverse Primer ( 5′ to 3′)
1	RP11‐469H8.6	tgaggtgatgggagaggtg	acctccttggggtaggtcat
2	LINC00281	aaagaagatgctgccaatgag	ggaagtgagttatttcagggtacg
3	BC045663	acaagccatggacaacagc	gcacatgttggagattgcac
4	RP11‐791G16.2	gcctgcctgttacattcctg	tgcaaccacgaactaattttctc
5	AC009948.5	gagtagcggtggctgaaga	ggtgccatcaagttccaaaa
6	DMKN	cagagcggagaggaaagcac	gcctcactgactttagagccag
7	PIEGO2	atggcctcagaagtggtgtg	atgtccttgcatcgtcgtttt
8	APOLD1	agagatgtaacccaactcgttca	caggggaaggtgcatcctc
9	ABI1	accagtcctgctaggcttg	actgttttctcgacttccacttc
10	SND1	cctgagcggcagatcaacc	aggtagatcatgccatactctcg
11	TMED2	catcgacgtggagattacagg	ggtggacatccggttactaaaac
12	SCAF8	gtgcgacaatcccgacatca	tccccagggcaacgatataaa
13	SNX10	cacttttgctttcagatagcagc	acacacgcctcaatgtcttct

### 2.3 Microarray profiling

We established an expression profile of 30,584 human lncRNAs and 26,106 protein‐coding transcripts using the Arraystar Human LncRNA Microarray V3.0. Three replicates were used for pravastatin‐treated and vehicle‐treated groups. Total RNAs were amplified and transcribed into fluorescent complimentary RNA (cRNAs) by the Arraystar Flash RNA Labeling Kit (Arraystar). One microgram of each cRNA was labeled and hybridized onto the microarray slide using the Agilent Array platform. Hybridized arrays were washed and fixed before scanning with the Agilent DNA Microarray Scanner (Product#G2505C).

### 2.4 Array analysis

The Agilent Feature Extraction software (version 11.0.1.1) was used to analyze the images acquired from scanning the hybridized array. Quantile normalization was performed using the GeneSpring GX v11.5.1 software package (Agilent Technologies) and *p*‐values were calculated for the differentially expressed genes by student's *t* test. *p‐*values were then subjected to multiple testing by the Benjamini Hochberg method for minimizing false discovery rate. Volcano‐plot filtering set at a threshold of ≥2.0‐folds was used to screen the lncRNAs and mRNAs exhibiting significantly different expression levels between the two experimental groups (adjusted *p* < .05; unpaired *t* test). Pathway analyses were conducted using the current Kyoto Encyclopedia of Genes & Genomes (KEGG) database.

## 3 RESULTS

### 3.1 Quality and expression assessment of lncRNAs and mRNAs

We first evaluated the integrity and purity of the total RNAs extracted from HUVECs. Employing the standard denaturing agarose gel electrophoresis, RNAs from all samples showed an upper 28S ribosomal RNA band that was twice as intense compared to the lower 18S band, validating the integrity of our RNA extracts (Supp. Figure S1). RNA purity was also verified also by these upper 28S ribosomal RNA bands presenting without any surrounding smears, as well as by the optical density (OD) A260/A280 and A260/A230 ratios close to 2.0 and exceeding 1.8, respectively, obtained from the NanoDrop ND‐1000 (Singh, Adam, et al., 2020). Cluster analysis of the distribution of fluorescent intensities for lncRNAs and mRNAs demonstrated similar patterns in global gene expression between samples and groups across the board that is illustrated by box and whisker plot (10th, 90th percentile) (Supp. Figure S2).

**Figure 1:**
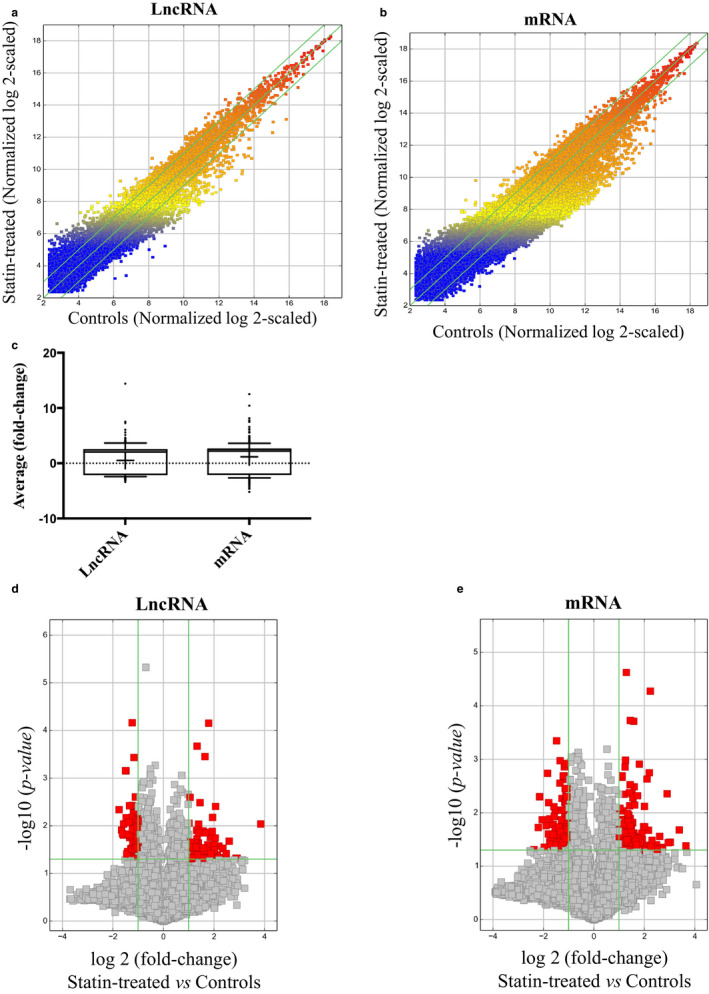
LncRNA and mRNA expression profiles in HUVECs treated with pravastatin (10 µM) versus control. (a and b) Scatter plots comparing the variation in lncRNA and mRNA expression. The values plotted are the averaged normalized signal values (log2‐scaled) for the control (x‐axis) and the pravastatin‐treatment (y‐axis) groups. The green lines indicate fold‐change. LncRNAs and mRNAs above the top green line and below the bottom green line exhibit at least a 2.0‐fold difference between the two study groups. (c) Box‐and‐Whisker plots (10th, 90th percentile) showing average fold‐change of lncRNAs and mRNAs. Median intensity is denoted with a “‐” and mean intensity denoted with a “+” sign. (d & e) Volcano plots detailing the magnitude of expression difference. The vertical green lines correspond to 2.0‐fold upregulation and 2.0‐fold downregulation of expression. The horizontal green line indicates an adjusted p‐value of ≤ 0.05. Red points represent lncRNAs and mRNAs with statistically significant differential expression (fold‐change ≥ 2.0, adjusted *p* ≤ .05)

Our analysis presented a profile of significantly upregulated and downregulated lncRNAs (Figure 1a) and mRNAs (Figure 1b) in response to pravastatin in HUVECs. We also examined the differential expression degree and observed similar average fold‐changes between lncRNAs and mRNAs (Figure 1c). From this profile, we uncovered 95 significantly upregulated and 86 significantly downregulated lncRNAs in pravastatin‐treated HUVECs compared to controls (Figure 1d), of which the lncRNAs ranged from 10,350 to 156 bp in size. Particularly, LINC00281 (RNA length: 3,215 bp, chromosome 22) and BC045663 (RNA length: 1823 bp, chromosome 20) in pravastatin‐treated HUVECs were the most upregulated (~8‐fold) and downregulated (~3‐fold) lncRNAs, respectively (Tables 2 and 3). We validated our findings by performing qPCR for selected upregulated lncRNAs LINC00281 and RP11‐469H8.6, and downregulated lncRNAs BC045663, RP11‐791G16.2, and AC009948.5. Among upregulated lncRNAs, RP11‐469H8.6 was 4.42 ± 1.48 (*p* = .0021) fold upregulated in the pravastatin‐treated group; however, qPCR for LINC00281 did not provide quantifiable data due to extremely low expression in the control group. Validation for all selected downregulated lncRNAs [BC045663 (0.288 ± 0.177‐fold, *p* = .0018), RP11‐791G16.2 (0.399 ± 0.008‐fold, *p* = .0008) and AC009948.5 (0.669 ± 0.050‐fold, *p* = .01850)] showed similar patterns as observed in the lncRNA array (Table 4). At the mRNA level, 190 differentially expressed mRNAs were upregulated, while 90 differentially expressed mRNAs were downregulated (Figure 1e) in HUVECs in response to pravastatin treatment, with the endocytosis‐associated protein DMKN (Dermokine) and cell growth‐associated ABI1 (ABL Interactor 1) (Biesova et al., 1997) being the most upregulated (~11‐fold) and downregulated (~ 5‐fold) gene, respectively (Tables 5 and 6). Validation qPCR performed for selected upregulated (DMKN, PIEGO2, and APOLD1) or downregulated (AB1I, SND1, TMED2, SCAF8, and SNX10) genes demonstrated a similar expression profile as observed in the array. Among upregulated genes, DMKN (5.49 ± 1.19‐fold, *p* = .001), PIEGO2 (1.755 ± 0.428‐fold, *p* = .033) and APOLD1 (1.296 ± 0.179‐fold, *p* = .020) were significantly upregulated genes in the pravastatin‐treated group in comparison to the control group. Similarly, among downregulated genes, AB1I (0.643 ± 0.179, *p* = .044), SCAF8 (0.728 ± 0.114, *p* = .028) and SNX10 (0.536 ± 0.313, *p* = .022) were significantly downregulated in the pravastatin‐treated group, but SND1 (0.813 ± 0.431, *p* = .436) and TMED2 (1.174 ± 0.367, *p* = .344) appeared to be unaffected by pravastatin in our validation qPCR (Table 4). As a supplement, we also provide the complete array data on our differentially expressed mRNAs and lncRNAs, their respective sequences, exact chromosomal locations, fold changes, and known target genes in Supp. Table S1 (https://figshare.com/s/82bb5cdf645b549ae416).

**Table 2:** Ten most upregulated lncRNAs in HUVECs upon pravastatin (10 µM) stimulation in comparison to controls

Top 10 upregulated differentially expressed lncRNAs in Statin‐treated versus Control endothelial cells						
Nr	Gene symbol	Fold change	*Adj. p‐value*	Associated gene	RNA length	Chr/ Strand
1	LINC00281	7.5390834	.0476663		3,215	22/−
2	AK125078	7.2998619	.0477598		847	1/‐
3	XLOC_007368	6.0619879	.0209871		218	9/+
4	XLOC_004244	5.5920067	.0383514		690	5/+
5	RP11‐469H8.6	5.1033278	.0296308	AQP2	592	12/‐
6	RP11‐493L12.2	4.5832587	.046438631	PCED1B	359	12/‐
7	RP11‐983C2.2	4.5736328	.026541316		482	16/‐
8	RP11‐473M20.9	4.3979907	.04118165	IL32	1,207	2/‐
9	XLOC_001747	4.1675991	.003911454		383	5/+
10	XLOC_005737	4.0376803	.032008961	RUNX2	2,243	6/‐

Abbreviations: Adj, Adjusted; AQP2, Aquaporin 2; IL32, Interleukin 32; PCED1B, PC‐Esterase Domain Containing 1B; RUNX2, Runt‐related Transcription Factor 2.

**Table 3:** Ten most downregulated lncRNAs in HUVECs upon pravastatin (10 µM) stimulation in comparison to controls

Top 10 downregulated differentially expressed lncRNAs in Statin‐treated versus Control endothelial cells						
Nr	Gene symbol	Fold change	*Adj. p‐value*	Associated gene	RNA length	Chr/ Strand
1	BC045663	3.3799237	.0045743		1823	20/‐
2	RP11‐876N24.2	3.1491515	.0122195	CIITA	575	16/‐
3	XLOC_001373	3.0753019	.0146288		387	2/+
4	RP11‐791G16.2	3.0002025	.0156631	TMEM150C	1,493	4/‐
5	RPS11P6	2.9800209	.0091166	C12orf56	1,352	12/+
6	XLOC_008645	2.8372396	.000696542		310	10/+
7	UTY	2.8021482	.00069716	UTY	6,761	Y/‐
8	AC009948.5	2.7780046	.00671064	PRKRA	563	2/+
9	REV3L‐IT1	2.7320222	.038811723	REV3L	382	6/‐
10	RP11‐542B15.1	2.6786082	.034661639		559	12/+

Abbreviations: Adj, Adjusted; C12orf56, Chromosome 12 Open Reading Frame 56; CIITA, Major Histocompatibility Complex Class II, Transactivator; PRKRA, Protein Kinase, Interferon‐Inducible Double‐Stranded RNA‐Dependent Activator; REV3L, Rev3, S. Cerevisiae, Homolog Of; TMEM150C, Transmembrane Protein 150C; UTY, Ubiquitously Transcribed Tetratricopeptide Repeat Gene on Y Chromosome.

**Table 4:** Validation was performed for selected top up or downregulated lncRNAs and mRNAs isolated from HUVECs upon pravastatin (10 µM) stimulation and controls

Nr	Gene symbol	Array Data (Fold change)	*p‐value* (Adj)	qPCR Data (Fold change)	*p*‐value
1	RP11‐469H8.6	5.1033278	.0296308	4.42 ± 1.48	.002
2	BC045663	−3.3799237	.0045743	0.288 ± 0.177	.001
3	RP11‐791G16.2	−3.0002025	.0156631	0.399 ± 0.008	.000
4	AC009948.5	−2.7780046	.00671064	0.669 ± 0.050	.018
5	DMKN	10.4331054	.020871174	5.49 ± 1.19	.001
6	PIEGO2	7.7281947	.036009804	1.755 ± 0.428	.033
7	APOLD1	7.4822837	.00441241	1.296 ± 0.179	.020
8	ABI1	−4.6038017	.018848386	0.643 ± 0.179	.044
9	SND1	−4.4388801	.004988181	0.813 ± 0.431	.436
10	TMED2	−4.135675	.013520699	1.174 ± 0.367	.344
11	SCAF8	−3.7269421	.017383289	0.728 ± 0.114	.028
12	SNX10	−3.566869	.001821224	0.536 ± 0.313	.022

Note

The “‐” sign indicates downregulation and qPCR data are presented as mean ± *SD*.

**Table 5:** Ten most upregulated mRNAs in HUVECs upon pravastatin (10 µM) stimulation in comparison to controls

Top 10 upregulated differentially expressed mRNAs in Statin‐treated versus Control endothelial cells			
Nr	Gene symbol	Fold change	*Adj p‐value*
1	DMKN	10.4331054	.020871174
2	TNNT2	8.1375169	.035583326
3	PIEZO2	7.7281947	.036009804
4	APOLD1	7.4822837	.00441241
5	C7orf34	6.5808857	.044510939
6	EIF2B4	6.1793913	.045905061
7	TMEM191B	6.0818645	.027576263
8	ENTHD2	5.685957	.047950255
9	A1CF	5.5672248	.03958813
10	LOC100287177	4.9306594	.032827533

Abbreviations: A1CF, Apobec1 Complementation Factor; Adj, Adjusted; APOLD1, Apolipoprotein L Domain‐Containing 1; C7orf34, Chromosome 7 Open Reading Frame 34; DMKN, Dermokine; EIF2B4, Eukaryotic Translation Initiation Factor 2b, Subunit 4; PIEZO2, Piezo‐Type Mechanosensitive Ion Channel Component 2; TMEM191B, Transmembrane Protein 191B; TNNT2, Troponin T2, Cardiac.

**Table 6:** Ten most downregulated mRNAs in HUVECs upon pravastatin (10 µM) stimulation in comparison to controls

Top 10 downregulated differentially expressed mRNAs in Statin‐treated versus Control endothelial cells			
Nr	Gene symbol	Fold change	*Adj. p‐value*
1	ABI1	4.6038017	.018848386
2	SND1	4.4388801	.004988181
3	TMED2	4.135675	.013520699
4	RAB23	3.9035519	.044063826
5	SCAF8	3.7269421	.017383289
6	SNX10	3.566869	.001821224
7	EMD	3.4986437	.024932582
8	SGK1	3.4659766	.006466557
9	B4GALT6	3.3686982	.013007055
10	PRIM2	3.3550803	.035131682

Abbreviations: ABI1, Abl Interactor 1; Adj, Adjusted; B4GALT6, Udp‐Gal:Beta‐Glcnac Beta‐1,4‐Galactosyltransferase, Polypeptide 6; EMD, Emerin; PRIM2, Primase Polypeptide 2a; RAB23, Ras‐Associated Protein Rab23; SCAF8, Sr‐Related C‐Terminal Domain‐Associated Factor 8; SGK1, Serum/Glucocorticoid‐Regulated Kinase 1; SND1, Staphylococcal Nuclease Domain‐ And Tudor Domain‐Containing Protein 1; SNX10, Sorting Nexin 10; TMED2, Transmembrane emp24 Domain‐containing Protein 2

### 3.2 LncRNA chromosomal distribution and subtype analysis

**Figure 2:**
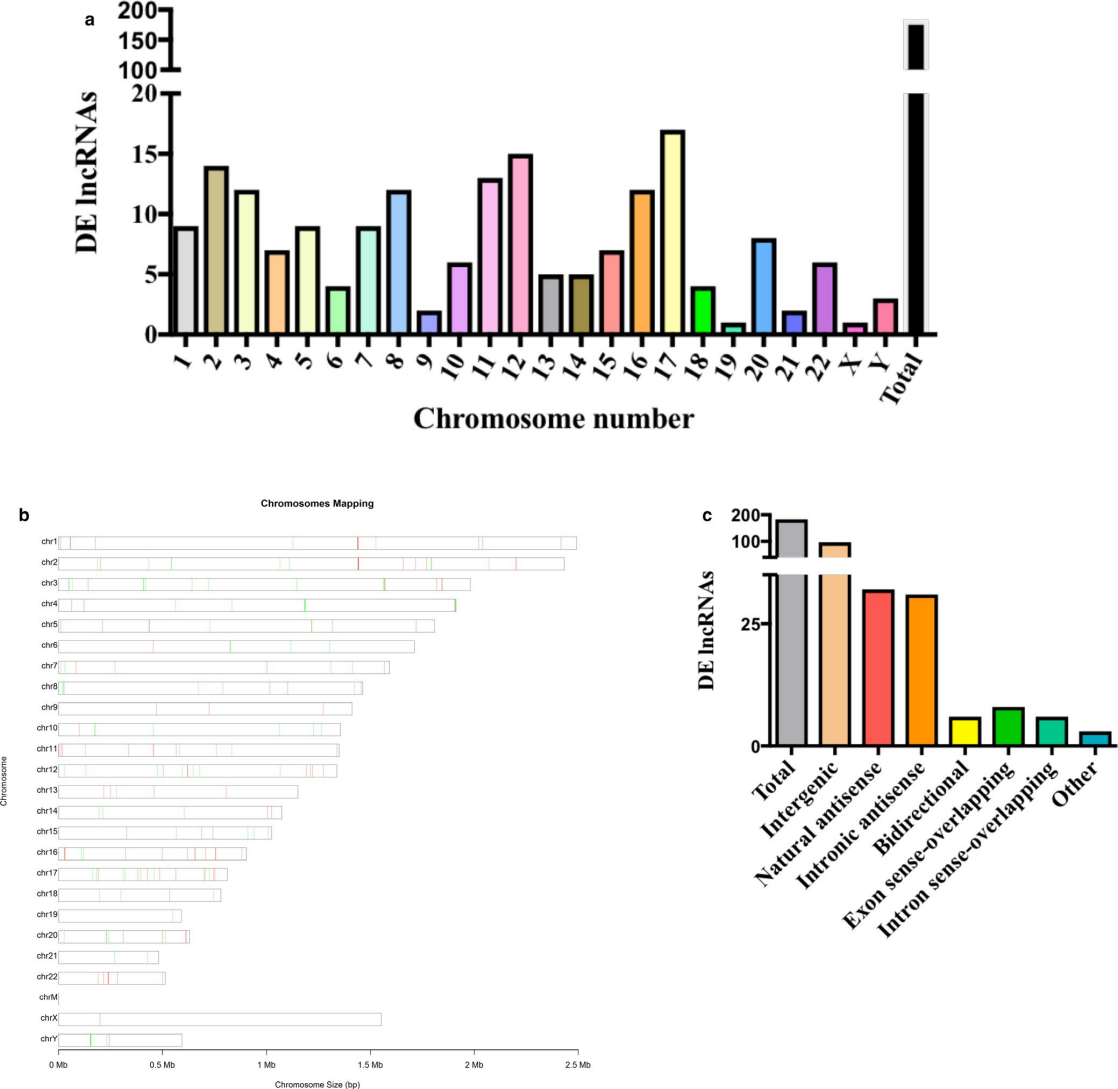
Distribution, location, and classification of differentially expressed lncRNAs in HUVECs treated with pravastatin (10 µM) versus control. Demonstration of (a) numbers and (b) chromosomal location of differentially expressed (DE) lncRNAs on different chromosomes. (c) Bar‐graph representing types of differently expressed lncRNAs, depending upon their genomic location

We proceeded to further examine the lncRNAs profile of pravastatin‐treated HUVECs with respect to the vehicle. After evaluating every chromosome, pravastatin‐induced differentially expressed lncRNAs were most abundant on chromosomes 2, 12, and 17 (Figure 2a). These lncRNAs were expressed along the entire length of the chromosomes with a notable clustering pattern (Figure 2b). LncRNAs are generally classified into six different categories based on the origin of their transcription and the arrangement of their neighboring genes. These categories are sense, natural antisense, intronic, intergenic, bidirectional promoter, and enhancer lncRNA. Subgroup analysis revealed that pravastatin‐induced differentially expressed lncRNAs were intergenic, bidirectional, natural and intronic antisense, exon‐ and intron sense‐overlapping. The majority were functionally intergenic in origin, followed by natural and intronic antisense (Figure 2c).

### 3.3 Bioinformatics analyses

To functionally associate the pravastatin‐induced differentially expressed lncRNAs, pathway analysis using the current KEGG database was conducted on the concomitant pravastatin‐induced differentially expressed mRNAs. We observed that in response to pravastatin, the genes upregulated in HUVECs were associated with the Rap1‐signaling pathway and osteoclast differentiation (Table 7), while those downregulated are involved in pathways noted in cancer and infection (Table 8).

**Table 7:** Results of bioinformatics analyses on upregulated pathways in HUVECs after pravastatin (10 µM) stimulation in comparison to controls

Upregulated pathways in Statin‐treated versus Control endothelial cells				
Nr	Pathways	Count	*Adj. p‐value*	Genes
1	Rap1 signaling pathway	5	.03996061	*FLT4//LCP2//PRKCI//RAP1GAP//VEGFB*
2	Osteoclast differentiation	4	.02855664	*FOSB//LCP2//LILRB1//OSCAR*
3	Maturity onset diabetes of the young	2	.02041033	*FOXA3//MNX1*

Abbreviation: Adj, Adjusted.

**Table 8:** Results of bioinformatics analyses on downregulated pathways in HUVECs after pravastatin (10 µM) stimulation in comparison to controls

Downregulated pathways in Statin‐treated versus Control endothelial cells				
Nr	Pathways	Count	*Adj. p‐value*	Genes
1	Pathways in cancer	5	.04629817	*CCDC6//JAK1//KIT//TRAF6//XIAP*
2	Epstein‐Barr virus infection	4	.03368377	*EIF2AK4//JAK1//SND1//TRAF6*
3	Hematopoietic cell‐lineage	3	.01593718	*IL5//ITGA1//KIT*
4	Neurotrophin signaling pathway	3	.03571419	*IRAK3//RPS6KA2//TRAF6*
5	Toxoplasmosis	3	.03571419	*JAK1//TRAF6//XIAP*
6	Hepatitis C	3	.0461964	*EIF2AK4//JAK1//TRAF6*
7	Measles	3	.04705986	*EIF2AK4//JAK1//TRAF6*
8	Ubiquitin mediated proteolysis	3	.04969846	*TRAF6//UBE3A//XIAP*
9	Autoimmune thyroid disease	2	.04216142	*IL5//TSHR*
10	NOD‐like receptor signaling pathway	2	.04649291	*NOD2//TRAF6*

Abbreviations Adj, Adjusted.

## 4 DISCUSSION

LncRNAs have been shown to exhibit multiple functions within the cell by modulating gene expression through their interactions with a diverse array of RNA and proteins. A number of lncRNAs have been implicated in the regulation of endothelial function such as endothelial‐to‐mesenchymal transition (Neumann et al., 2018), migration, proliferation, apoptosis (Man et al., 2018), hypoxia‐induced angiogenesis (Voellenkle et al., 2016), and regulation of eNOS expression (Cheng et al., 2019). Despite the growing body of work on lncRNAs in cardiovascular disease, their role in regulating endothelial function in response to lipid‐lowering therapy by statins is unknown. Previous studies have evaluated the effect of statins on individual lncRNAs such as LASER in hepatocytes and RP1‐13D10.2 in lymphoblastoid cells (Li et al., 2019; Mitchel et al., 2016). However, to our knowledge, this is the first study highlighting the differential expression of endothelial lncRNAs and mRNAs following statin treatment.

Using a dose of 10 μM pravastatin (Abe et al., 2006; Panczel et al., 2019), we were able to profile all differentially expressed mRNAs and lncRNAs in human ECs *in vitro*. A total of 181 lncRNAs and 280 mRNAs were significantly differentially expressed in pravastatin‐treated versus control ECs (log 2‐fold > 2, adjusted *p* < .05). Of the 30,854 lncRNAs, 95 were upregulated, and 86 were downregulated following pravastatin treatment. Validation qPCR performed for the selected four out of five most up‐ or downregulated lncRNAs demonstrated similar expression patterns in pravastatin‐treated in comparison to control ECs. In the same samples, 190 out of 280 differentially expressed mRNAs were upregulated and 90 mRNAs were downregulated. Validation qPCR performed for six out of eight selected most up‐ or downregulated genes confirmed a similar expression profile in the pravastatin‐treated ECs. The overall expression of lncRNAs was lower than mRNAs, which is consistent with other studies (Chen et al., 2018). Differentially expressed lncRNA and their class distribution were in order with other previously published reports on endothelial lncRNAs, with the majority being intergenic in nature (Singh, Mantella, et al., 2016; Singh, Matkar, Muhammad, et al., 2016; Singh et al., 2017; Singh, Matkar, Quan, et al., 2016). All the differentially regulated lncRNAs were evenly distributed on all chromosomes; however, it was interesting to note that five differentially expressed lncRNAs among the top 10 differentially upregulated and downregulated lncRNAs were located on chromosome 12, which has been previously linked by the whole‐genome scan with prevention and treatment of CVDs (Ganesh et al., 2013; Gong et al., 2003). Our data on chromosomal distribution and location will be of immense interest to geneticists as it provides a base to identify associated lncRNAs if present in the linked locus of the genome‐wide association study.

In our array data, most of the pravastatin‐induced significantly differentially expressed lncRNAs are novel and have not been characterized. However, based on prediction analyses and other relevant studies, we can hypothesize their potential roles in statin‐mediated changes and EC biology. The most significantly upregulated lncRNAs include XLOC_007368, XLOC_004244, RP11‐469H8.6, and RP11‐493L12.2 among others. LncRNA XLOC_007368 is predicted to have a binding site for microRNA 22‐3p, (Paraskevopoulou et al., 2016) which inhibits endothelial inflammation *in vitro* by targeting ICAM‐1, indicating a role for lncRNA XLOC_007368 in inflammation (Gidlof et al., 2015). LncRNA XLOC_004244 acts as a competitively endogenous RNA for the protein‐coding gene Acyl‐CoA Synthetase Long‐Chain Family Member 3 (ACSL3). ACSL3 belongs to a family of proteins that serve a key role in lipid biosynthesis and fatty acid degradation by converting free long‐chain fatty acids into Fatty Acyl‐CoA Esters (Li et al., 2010). Knockdown of ACSL3 inhibits fatty acid synthesis in primary hepatocytes (Bu et al., 2009) and its suppression perturbs fatty acid oxidation in cancer cells (Padanad et al., 2016). Although statins are known for the regulation of fatty acid metabolism, there are no studies linking statin use to direct changes in the ACSL3 expression. These findings highlight that the upregulation of XLOC_004244 would result in reduced ACSL3 protein expression leading to reduced fatty acid synthesis. LncRNA XLOC_004244 is also associated with the hsa‐miR‐485‐3p (Su et al., 2019). MiR‐485‐3p directly inhibits the expression of PGC‐1α to regulate mitochondrial respiration and cell migration (Lou et al., 2016). LncRNA RP11‐469H8.6 is predicted to target the expression of the aquaporin (AQP2) (Perron et al., 2017), which is known primarily for the regulation of water absorption *via* collecting duct principal cells, but little is known about AQP2 role in ECs (Kwon et al., 2013). This implies that statins may lead to a decrease in AQP2 expression in endothelial cells. LncRNA RP11‐493L12.2 expression is significantly downregulated in macrophages following mycobacterium tuberculosis and is thought to have a role in the regulation of macrophage apoptosis (Yang et al., 2016). However, its role in endothelial cells is unknown.

The most significantly downregulated lncRNAs include RP11‐876N24.2, XLOC_001373, and XLOC_008645, among others. RP11‐876N24.2 is located near the protein‐coding gene of class II MHC trans‐activator (CIITA). CIITA plays a key role in T cell‐mediated acute rejection following transplant as highlighted by the suppression of CD4 alloresponse following EC‐specific knockdown of CIITA (Abrahimi et al., 2016). The downregulation of RP11‐876N24.2 would, therefore, suggest increased CD4 alloresponse following statin use. LncRNA XLOC_001373, another downregulated lncRNA, has not been characterized but the role of its predicted interactor hsa‐miR‐345‐5p has been well studied in the cardiovascular system (Paraskevopoulou et al., 2016). MiR‐345‐3p directly targets TRAF6, thereby inhibiting oxLDL‐mediated apoptosis and inflammation in ECs by suppression of the TAK1/p38/NF‐kB signaling pathway (Wei et al., 2020). MiR‐345‐3p expression is also decreased in the apolipoprotein‐E deficient mouse model of atherosclerosis, further supporting this hypothesis (Chen et al., 2019). This is in line with statin‐mediated treatment as the downregulation of XLOC_001373 would lead to the upregulation of miR‐345‐3p leading to reduced inflammation in ECs. In addition, circulating miR‐345‐3p levels are elevated in early heart failure ( Wang et al., 2017), highlighting the potential role of XLOC_001373 in the cardiovascular system. LncRNA XLOC_008645 is predicted to interact with hsa‐miR‐127‐3p (Paraskevopoulou et al., 2016), which directly targets α6 Integrin (ITGA6). ITGA6 plays a key role in vascular endothelial growth factor‐A and fibroblast growth factor‐2‐driven angiogenesis (Primo et al., 2010). Therefore, this downregulation of XLOC_008645 would lead to a reduction in angiogenesis, which is relevant as statins, depending on their dose, have also been shown to inhibit and promote angiogenesis (Weis et al., 2002).

Transcriptome analyses following treatment with pravastatin revealed differential expressions of genes associated with endothelial function. The most upregulated genes included Dermokine (DMKN), Apolipoprotein L domain containing 1 (APOLD1), and Piezo‐Type Mechanosensitive Ion Channel Component 2 (PIEZO2), among others. DMKN, the most upregulated gene, promotes vascular endothelial growth factor (VEGF) production and is also associated with high‐density lipid (HDL) proteome within liver cells (Pamir et al., 2019; Pentecost & Yudkin, 1996). APOLD1 is highly expressed in ECs of developing tissues (Regard et al., 2004). Endothelial PIEZO2 plays a role in mechanosensing, which is required for the maintenance of vascular homeostasis (Zhong et al., 2018).

The most downregulated genes included ABL‐Interactor 1 (ABI1), Transmembrane p24 Trafficking Protein 2 (TMED2), and RAS‐Associated Protein 23 (RAB23), among others. ABI1 is critical for coordinating cytoskeletal organization and actin polymerization, and plays a role in cardiovascular development (Kotula, 2012; Ring et al., 2011). Reduced expression of TMED2, a protein involved in vesicular trafficking (Jerome‐Majewska et al., 2010), is in line with other findings where statins are shown to inhibit vesicular trafficking in other cell types (Sakamoto et al., 2011; Wade, 2011). RAB23 belongs to the superfamily of small GTPase, is a negative regulator of Sonic hedgehog signaling (Shh), and plays a critical role in the development of multiple organs (Eggenschwiler et al., 2001, 2006). The role of RAB23 in ECs is unknown; however, statins have been shown to inhibit Shh signaling in hepatic stellate cells. This is due to the cholesterol‐lowering ability of statins as Shh signaling is required for cholesterol biosynthesis (Gordon et al., 2018; Uschner et al., 2015). These findings suggest that Rab23 might represent an important signaling protein in regulating Shh signaling within ECs. Statin‐induced differentially expressed genes that are not characterized in ECs and have also not been studied in relation to statin, warrant further investigation. However, most of the observed changes do support the mechanisms by which statins exert their effect in ECs and other cell types.

Pathway analysis revealed upregulated Rap1 (Ras‐associated protein‐1) signaling pathway, osteoclast differentiation, and maturity‐onset diabetes of the young. Rap1 is a small GTPase that has diverse functionality regulating basic cellular functions such as cell adhesions and junctions, cell migration, and polarization (Zhang et al., 2017). In ECs, Rap1 plays a role in stabilizing newly formed vessels, promotes angiogenesis and endothelial barrier function, and is critical for NO production, therefore, controls the vascular tone and stabilizes blood pressure (Chrzanowska‐Wodnicka et al., 2008; Lakshmikanthan et al., 2015; Yan et al., 2008). The impaired NO production in Rap1 EC‐KO mice is due to the inability of ECs to sense shear stress in the absence of Rap1. Interestingly, statins inhibit EC Rap1 activity in a dose‐dependent manner (Kou et al., 2012). While the actions of statins on the Rap1 pathway have been highlighted before, no functional studies to date have linked lncRNAs and the regulation of the Rap1 signaling pathway. The downregulated pathways after statin‐treatment in EC include pathways in cancer, Epstein‐Barr virus infection, hematopoietic cell‐lineage, and neurotrophin signaling. The cancer‐associated pathway can be attributed to the overlap of cell regulatory genes and lncRNAs that are common to both cardiovascular diseases and cancer. The linkage of the downregulated genes to infection‐related pathways is in line with statin treatment. Statins have been shown to have an anti‐inflammatory effect on ECs as they inhibit the expression of cell adhesion molecules intercellular adhesion molecule‐1 (ICAM‐1) (Chung et al., 2002), vascular cell adhesion molecule (VCAM‐1), and E‐selectin (Rasmussen et al., 2001). While there is not much available information on other significant up‐ or downregulated pathways in the endothelium, our data provide the first hint toward the significant roles of these pathways in the endothelium, particularly after statin‐treatment, and warrant future investigation.

There are some limitations to the present work. Although HUVECs are an established representative cell type for endothelial research *in vitro*, it is important to validate our findings in other EC types, such as in human coronary artery ECs and human microvascular ECs to confirm the non‐specificity of our findings. Our conclusions cannot be generalized for all statins because we only used pravastatin and different statins can have varying therapeutic and toxic effects both *in vitro* and *in vivo* (Ward et al., 2019). One of the strengths, which is also a weakness in our report, is the novelty since most of the identified differentially expressed lncRNA are not characterized; therefore, it is difficult to associate the relevance of these changes with pravastatin and the endothelium. Thus, we strongly recommend further characterization of these novel lncRNAs.

In conclusion, the present study is the first, to our knowledge, to demonstrate the expression profile of lncRNAs and mRNAs in ECs following treatment with pravastatin. We identified several lncRNAs that have previously not been associated with signaling pathways in the endothelium and therefore present as novel targets. Our bioinformatic analyses shed light on some of the pathways that might govern endothelial function following pravastatin treatment. Our findings identify novel biomarkers and potential therapeutic targets in addition to providing insights into the mechanisms underlying the effects of pravastatin in ECs. Future investigations in these directions are warranted.

## CONFLICTS OF INTEREST

None.

## AUTHOR CONTRIBUTIONS

KS conceived and designed the study. KS and HN carried out the experiments and analyzed the data. SS, HN, ME, DM, and MQ helped improve the discussion. SS, HN, PS, ME and KS wrote, assembled, and revised the manuscript with final figures.

## Supporting information



Fig S1‐S2
