# Supplementary material for: Pravastatin‐induced changes in expression of long non‐coding and coding RNAs in endothelial cells
Source: Physiol Rep. 2020 Dec 28;9(1):e14661. doi: 10.14814/phy2.14661 (PMC7769171; doi:10.14814/phy2.14661)
Supplement: Supplementary file 1 — Fig S1‐S2 [file PHY2-9-e14661-s001.pdf]

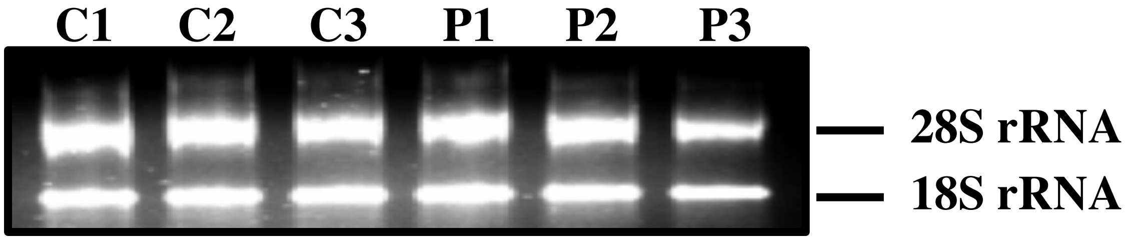

Supplementary Figure 1. Quality Assessment of RNA Samples. Denaturing agarose gel (0.5%) electrophoresis was performed using 900 ng of RNA (500 ng for P3) to assess RNA integrity and genomic DNA contamination. The gel image shows clear and intact 28S and 18S rRNA bands. C1, C2 and C3 correspond to three control samples. P1, P2 and P3 correspond to three pravastatin-treated (10 μM) samples.

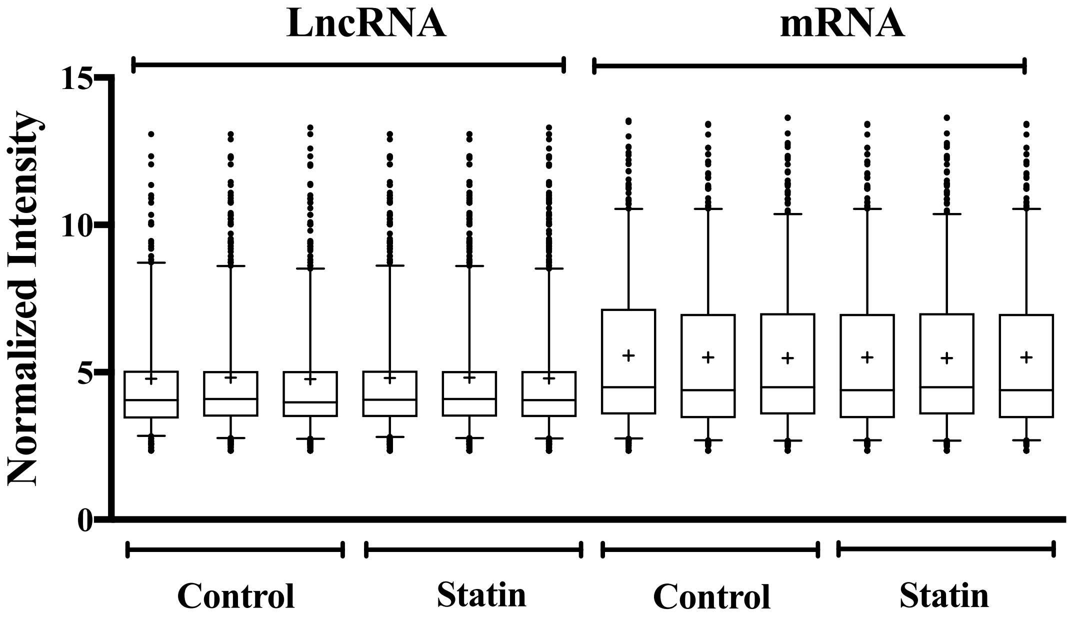

Supplementary Figure 2. Quality assessment of lncRNAs and mRNAs Data. Box-and-Whisker plots (10th, 90th percentile) showing normalized intensity for the 6 study samples to quickly visualize the distribution of our dataset. Mean intensity is denoted with a "+" sign. Control represents vehicle-treated and Statin represents pravastatin-treated (10 μM) group.
